# Supplementary material for: Polyketide synthase gene clusters in the ericoid mycorrhizal fungus Oidiodendron maius: genome-wide prediction, expression profiling, and functional characterization of knockout mutants
Source: Front Plant Sci. 2026 Sep 16;17:1924430. doi: 10.3389/fpls.2026.1924430 (PMC13623903; doi:10.3389/fpls.2026.1924430)
Supplement: Supplementary file 1 [file SupplementaryFile1.zip › Supplementary Materials/Supplementary Folder 1.zip!/Oidma1_AssemblyScaffolds_Repeatmasked/knownclusterblast/cluster47/jgi.p_Oidma1_106796_mibig_hits.html]

| MIBiG Protein | Description | MIBiG Cluster | MiBiG Product | % ID | % Coverage | BLAST Score | E-value |
| --- | --- | --- | --- | --- | --- | --- | --- |
| AMR44276.1 | MFS\_multidrug\_transporter | BGC0001398\_c3 | Phomopsins\_biosynthetic\_gene\_cluster | 76 | 102.608695652 | 872 | 3.3e-253 |
| AAS90069.1 | AflT | BGC0000010 | Aflatoxin\_biosynthetic\_gene\_cluster | 53 | 90.2608695652 | 531 | 1.4e-150 |
| AAS89998.1 | AflT | BGC0000007 | Aflatoxin\_biosynthetic\_gene\_cluster | 53 | 90.2608695652 | 525 | 5.8e-149 |
| AAS90046.1 | AflT | BGC0000009 | Aflatoxin\_biosynthetic\_gene\_cluster | 52 | 91.8260869565 | 523 | 2.9e-148 |
| AAS90092.1 | AflT | BGC0000006 | Aflatoxin\_biosynthetic\_gene\_cluster | 53 | 90.2608695652 | 519 | 5.4e-147 |
| AAS90021.1 | AflT | BGC0000008 | Aflatoxin\_biosynthetic\_gene\_cluster | 52 | 90.2608695652 | 517 | 2.7e-146 |
| BAE71313.1 | putative\_ABC\_transporter | BGC0000004 | Aflatoxin\_biosynthetic\_gene\_cluster | 55 | 79.3043478261 | 503 | 3.1e-142 |
| ADY16699.1 | TqaJ | BGC0001142 | Tryptoquialanine\_biosynthetic\_gene\_cluster | 50 | 87.4782608696 | 482 | 7.3e-136 |
| CAP96439.1 |  | BGC0000420 | Roquefortine\_C\_/\_meleagrin\_biosynthetic\_gene\_cluster | 50 | 85.9130434783 | 472 | 5.8e-133 |
| ACD39756.1 | major\_facilitator\_superfamily\_transporter | BGC0000076 | Hypothemycin\_biosynthetic\_gene\_cluster | 49 | 91.4782608696 | 471 | 1.3e-132 |
| ACD39765.1 | major\_facilitator\_superfamily\_transporter | BGC0000077 | Hypothemycin\_biosynthetic\_gene\_cluster | 49 | 91.4782608696 | 471 | 1.3e-132 |
| BAD29973.1 | transporter\_protein | BGC0000676 | Aphidicolin\_biosynthetic\_gene\_cluster | 46 | 89.9130434783 | 461 | 1.7e-129 |
| CBF87265.1 | MFS\_toxin\_efflux\_pump\_(AflT),\_putative\_(AFU\_orthologue;\_AFUA\_1G12620) | BGC0000680\_c2 | Austinol/dehydroaustinol,\_locus\_1\_biosynthetic\_gene\_cluster | 41 | 103.130434783 | 441 | 1.1e-123 |
| XP\_002384861.1 | MFS\_multidrug\_transporter,\_putative | BGC0001234\_c2 | 4,4'-piperazine-2,5-diyldimethyl-bis-phenol\_biosynthetic\_gene | 41 | 84.0 | 406 | 6.6e-113 |
| CCE28984.1 | probable\_DHA14-like\_major\_facilitator;\_ABC\_transporter | BGC0001365 | Clapurines\_biosynthetic\_gene\_cluster | 42 | 90.6086956522 | 402 | 7.3e-112 |
| AAB36607.1 | toxin\_pump | BGC0000370\_c2 | HC-toxin\_biosynthetic\_gene\_cluster | 39 | 93.0434782609 | 370 | 4e-102 |
| ACZ66257.1 | APS11 | BGC0000304 | Apicidin\_biosynthetic\_gene\_cluster | 40 | 90.6086956522 | 366 | 4.5e-101 |
| AHV78251.1 | ResE | BGC0001246 | Trans-resorcylide\_biosynthetic\_gene\_cluster | 39 | 80.6956521739 | 332 | 7.1e-91 |
| ADM34144.1 | efflux\_pump | BGC0001084 | Notoamide\_/\_stephacidin\_biosynthetic\_gene\_cluster | 39 | 85.9130434783 | 331 | 1.6e-90 |
| AAD34558.1 | unknown | BGC0000089 | Lovastatin\_biosynthetic\_gene\_cluster | 40 | 86.2608695652 | 331 | 2.1e-90 |
| ABA02247.1 | efflux\_pump | BGC0000098 | Monacolin\_K\_biosynthetic\_gene\_cluster | 40 | 89.5652173913 | 328 | 1.8e-89 |
| ADI24949.1 | GsfJ | BGC0000070 | Griseofulvin\_biosynthetic\_gene\_cluster | 40 | 80.5217391304 | 326 | 8.7e-89 |
| CBF76046.1 | conserved\_hypothetical\_protein | BGC0001399 | Fellutamide\_B\_biosynthetic\_gene\_cluster | 39 | 88.347826087 | 326 | 8.7e-89 |
| BAC20568.1 | efflux\_pump | BGC0000039 | Compactin\_biosynthetic\_gene\_cluster | 39 | 89.5652173913 | 325 | 1.1e-88 |
| AGC95323.1 | CurE | BGC0000045 | Dehydrocurvularin\_biosynthetic\_gene\_cluster | 35 | 94.0869565217 | 317 | 2.4e-86 |
| EAU36749.1 | predicted\_protein | BGC0000292 | Acetylaranotin\_biosynthetic\_gene\_cluster | 40 | 83.4782608696 | 302 | 1.4e-81 |
| ctg1\_orf0002 |  | BGC0000688 | Copalyl\_diphosphate\_biosynthetic\_gene\_cluster | 38 | 73.7391304348 | 273 | 6.7e-73 |
| ACS68556.1 | major\_facilitator\_superfamily\_protein | BGC0001026 | NG-391\_biosynthetic\_gene\_cluster | 31 | 84.347826087 | 235 | 1.2e-61 |
| CAF31575.1 | putative\_kanamycin\_efflux\_protein | BGC0000705 | Kanamycin\_biosynthetic\_gene\_cluster | 34 | 81.2173913043 | 227 | 3.2e-59 |
| CAF60521.1 | putative\_efflux\_protein | BGC0000704 | Kanamycin\_biosynthetic\_gene\_cluster | 34 | 81.2173913043 | 227 | 3.2e-59 |
| EAQ86390.1 | hypothetical\_protein | BGC0001405 | Chaetoviridin\_/\_chaetomugilin\_biosynthetic\_gene\_cluster | 31 | 75.3043478261 | 225 | 1.6e-58 |
| ACZ57546.1 | predicted\_MFS\_transporter | BGC0000046 | Depudecin\_biosynthetic\_gene\_cluster | 31 | 88.5217391304 | 220 | 5.2e-57 |
| EAA65599.1 | hypothetical\_protein | BGC0000022 | Asperfuranone\_biosynthetic\_gene\_cluster | 31 | 93.5652173913 | 220 | 6.7e-57 |
| AAF00219.1 | transporter | BGC0000276 | Urdamycin\_biosynthetic\_gene\_cluster | 31 | 85.9130434783 | 211 | 3.1e-54 |
| BAE56594.1 |  | BGC0001123 | Aspirochlorine\_biosynthetic\_gene\_cluster | 31 | 76.1739130435 | 200 | 7.2e-51 |
| AMY15055.1 | MFS\_transporter | BGC0001339 | Squalestatin\_S1\_biosynthetic\_gene\_cluster | 27 | 87.652173913 | 195 | 1.8e-49 |
| AHW57792.1 | PgaJ3 | BGC0000262 | Rabelomycin\_biosynthetic\_gene\_cluster | 29 | 75.1304347826 | 190 | 5.7e-48 |
| AFU65888.1 | DacR2 | BGC0000216 | Dactylocycline\_biosynthetic\_gene\_cluster | 31 | 74.2608695652 | 184 | 3.1e-46 |
| AGO50605.1 | transporter | BGC0000229 | Grincamycin\_biosynthetic\_gene\_cluster | 30 | 88.8695652174 | 181 | 2e-45 |
| AHA12095.1 | transporter | BGC0001172 | Chlorizidine\_A\_biosynthetic\_gene\_cluster | 30 | 76.1739130435 | 174 | 4.2e-43 |
| AAL15595.1 | Sim17 | BGC0000270 | Simocyclinone\_biosynthetic\_gene\_cluster | 28 | 91.3043478261 | 162 | 1.7e-39 |
| AAK06799.1 | putative\_export\_protein\_SimEx | BGC0001072 | Simocyclinone\_biosynthetic\_gene\_cluster | 28 | 91.3043478261 | 160 | 8.3e-39 |
| AGA37273.1 | efflux\_pump | BGC0000819 | Paraherquamide\_biosynthetic\_gene\_cluster | 41 | 33.9130434783 | 152 | 1.3e-36 |
| ABC87523.1 | putative\_drug\_efflux\_transporter | BGC0001011 | Meridamycin\_biosynthetic\_gene\_cluster | 28 | 82.2608695652 | 138 | 2.6e-32 |
| EYT83437.1 | multidrug\_MFS\_transporter | BGC0001213 | Nataxazole\_biosynthetic\_gene\_cluster | 28 | 68.6956521739 | 124 | 3e-28 |
| ABC87516.1 | drug\_efflux\_transporter | BGC0001011 | Meridamycin\_biosynthetic\_gene\_cluster | 28 | 56.5217391304 | 100 | 6e-21 |
| CAH60162.2 | putative\_transmembrane\_efflux\_protein | BGC0000700 | Istamycin\_biosynthetic\_gene\_cluster | 26 | 74.0869565217 | 91 | 2.8e-18 |
| AEA60653.1 | transporter | BGC0000892 | Caryoynencin\_biosynthetic\_gene\_cluster | 24 | 74.2608695652 | 91 | 4.7e-18 |
